# Neural Population Dynamics Underlying Expected Value Computation

**DOI:** 10.1523/JNEUROSCI.1987-20.2020

**Published:** 2021-02-24

**Authors:** Hiroshi Yamada, Yuri Imaizumi, Masayuki Matsumoto

**Affiliations:** ^1^Division of Biomedical Science, Faculty of Medicine, University of Tsukuba, Tsukuba 305-8577, Ibaraki, Japan; ^2^Graduate School of Comprehensive Human Sciences, University of Tsukuba, Tsukuba 305-8577, Ibaraki, Japan; ^3^Transborder Medical Research Center, University of Tsukuba, Tsukuba 305-8577, Ibaraki, Japan; ^4^Medical Sciences, University of Tsukuba, Tsukuba 305-8577, Ibaraki, Japan

**Keywords:** computation, expected values, integration, monkey, neural population dynamics, rewards

## Abstract

Computation of expected values (i.e., probability × magnitude) seems to be a dynamic integrative process performed by the brain for efficient economic behavior. However, neural dynamics underlying this computation is largely unknown. Using lottery tasks in monkeys (*Macaca mulatta*, male; *Macaca fuscata*, female), we examined (1) whether four core reward-related brain regions detect and integrate probability and magnitude cued by numerical symbols and (2) whether these brain regions have distinct dynamics in the integrative process. Extraction of the mechanistic structure of neural population signals demonstrated that expected value signals simultaneously arose in the central orbitofrontal cortex (cOFC; medial part of area 13) and ventral striatum (VS). Moreover, these signals were incredibly stable compared with weak and/or fluctuating signals in the dorsal striatum and medial OFC. Temporal dynamics of these stable expected value signals were unambiguously distinct: sharp and gradual signal evolutions in the cOFC and VS, respectively. These intimate dynamics suggest that the cOFC and VS compute the expected values with unique time constants, as distinct, partially overlapping processes.

**SIGNIFICANCE STATEMENT** Our results differ from those of earlier studies suggesting that many reward-related regions in the brain signal probability and/or magnitude and provide a mechanistic structure for expected value computation employed in multiple neural populations. A central part of the orbitofrontal cortex (cOFC) and ventral striatum (VS) can simultaneously detect and integrate probability and magnitude into an expected value. Our empirical study on these neural population dynamics raises a possibility that the cOFC and VS cooperate on this computation with unique time constants as distinct, partially overlapping processes.

## Introduction

Economic behavior requires a reliable perception of the world for maximizing benefit (Von Neumann and Morgenstern, 1944; Houthakker, 1950; Samuelson, 1950; Savage, 1954). Such maximization is primarily achieved by computing expected values (EVs; i.e., probability multiplied by magnitude) in the brain (Glimcher et al., 2008), which seems to be a dynamic process for detecting and integrating probability and magnitude to yield expected value signals. Indeed, humans and animals behave as if they compute the expected values in the brain (Kahneman and Tversky, 1979; Stephens and Krebs, 1986; Glimcher et al., 2008). One salient example, discovered over a century ago and repeatedly measured, is human economic behavior, in which a series of models originating from the standard theory of economics (Von Neumann and Morgenstern, 1944) has been developed to describe efficient economic behavior. Despite the ubiquity of this phenomenon, a dynamic integrative process to compute the expected values from probability and magnitude remains largely unknown.

In the past 2 decades, substantial research in animals has suggested that various brain regions process rewards in terms of signaling probability and/or magnitude, mostly during economic choice behavior (Platt and Glimcher, 1999; Barraclough et al., 2004; Tobler et al., 2005; Roesch et al., 2009; Ma and Jazayeri, 2014; Rudebeck and Murray, 2014; Eshel et al., 2016; Lopatina et al., 2016; Xie and Padoa-Schioppa, 2016; Yamada et al., 2018). Among these, expected value computation is assumed to be processed by neurons in many regions without their neural dynamics, which is in line with the expected value theory shared across multiple disciplines (Von Neumann and Morgenstern, 1944; Stephens and Krebs, 1986; Sutton and Barto, 1998; Glimcher et al., 2008). Neuroimaging studies in humans and nonhuman primates also suggest that multiple brain regions in the reward circuitry (Haber and Knutson, 2010) are involved in this computational process (O'Doherty et al., 2004; Tom et al., 2007; Hsu et al., 2009; Levy and Glimcher, 2011; Howard et al., 2015; Howard and Kahnt, 2017; Papageorgiou et al., 2017; Fouragnan et al., 2019), although the underlying neural mechanism has not been elucidated because of the limited time resolution of current neuroimaging techniques (Goense and Logothetis, 2008; Milham et al., 2018). Many brain regions may employ expected value computation; however, none of these studies could capture and compare temporal aspects of neural activities regarding expected value computation in the multiple candidate brain regions. Thus, we tested the hypothesis that neural population dynamics within sub-second-order time resolutions (Churchland et al., 2012; Mante et al., 2013; Chen and Stuphorn, 2015; Murray et al., 2017; Takei et al., 2017) play a key role in expected value computation, that is, the detection and integration of probability and magnitude on multiple neural population ensembles.

We targeted reward-related cortical and subcortical structures of nonhuman primates (Haber and Knutson, 2010): the central orbitofrontal cortex [cOFC; the medial part of area 13 (13 M)], the medial orbitofrontal cortex (mOFC; area 14O), dorsal striatum (DS; the caudate nucleus), and ventral striatum (VS), all of which represent neural correlates of probability and/or magnitude during economic choice behavior. We dissociated the integrative process computing the expected values from a neural process generating a choice command, which is used during economic choices (Chen and Stuphorn, 2015; Rich and Wallis, 2016; Gardner et al., 2019; Yoo and Hayden, 2020), by recording the neural activity in a nonchoice situation; monkeys perceive expected values from a single numerical symbol composed of probability and magnitude. We then applied a recently developing mathematical approach, called state space analysis (Churchland et al., 2012; Mante et al., 2013; Chen and Stuphorn, 2015; Murray et al., 2017), to the multiple neuronal activities to test how expected value computation is processed within each of the four neural population ensembles on the order of 10^−2^ s time resolution. Our findings suggest that the cOFC and VS neural populations employ a common integrative computation of expected values from probability and magnitude as distinct and partially overlapping processes.

## Materials and Methods

### Subjects and experimental procedures

Two rhesus monkeys were used for this study (*Macaca mulatta*, SUN, 7.1 kg, male; *Macaca fuscata*, FU, 6.7 kg, female). All experimental procedures were approved by the Animal Care and Use Committee of the University of Tsukuba (protocol #H30.336) and were performed in compliance with the US Public Health Service *Guide for the Care and Use of Laboratory Animals*. Each animal was implanted with a head-restraint prosthesis. Eye movements were measured using a video camera system at 120 Hz. Visual stimuli were generated by a liquid crystal display at 60 Hz placed 38 cm from the face of the monkey when seated. The subjects performed the cued lottery task 5 d/week. The subjects practiced the cued lottery task for 10 months, after which they became proficient in choosing lottery options.

### Experimental design

#### Behavioral task

##### Cued lottery tasks

Animals performed one of the following two visually cued lottery tasks: single-cue task or choice task. The activity of neurons was recorded only during the single-cue task.

##### Single-cue task

At the beginning of each trial, the monkeys had 2 s to align their gaze to within 3° of a 1°-diameter gray central fixation target. After fixating for 1 s, an 8° pie chart providing information about the probability and magnitude of rewards was presented for 2.5 s at the same location as the central fixation target. The pie chart was then removed and 0.2 s later, 1 and 0.1 kHz tones of 0.15 s duration indicated reward and no-reward outcomes, respectively. The high tone preceded a reward by 0.2 s. The low tone indicated that no reward was delivered. The animals received a fluid reward, for which magnitude and probability were indicated by the green and blue pie charts, respectively; otherwise, no reward was delivered. An intertrial interval of 4–6 s followed each trial.

##### Choice task

At the beginning of each trial, the monkeys had 2 s to align their gaze to within 3° of a 1°-diameter gray central fixation target. After fixating for 1 s, two peripheral 8° pie charts providing information about the probability and magnitude of rewards for each of the two target options were presented for 2.5 s, at 8° to the left and right of the central fixation location. Gray 1° choice targets appeared at these same locations. After a 0.5 s delay, the fixation target disappeared, cueing saccade initiation. The animals were free to choose for 2 s by shifting their gaze to either target within 3° of the choice target. A 1 and 0.1 kHz tone of 0.15 s duration indicated reward and no-reward outcomes, respectively. The animals received a fluid reward indicated by the green pie chart of the chosen target, with the probability indicated by the blue pie chart; otherwise, no reward was delivered. An intertrial interval of 4–6 s followed each trial.

##### Payoff and block structure

Green and blue pie charts indicated reward magnitudes from 0.1 to 1.0 ml, in 0.1 ml increments, and reward probabilities from 0.1 to 1.0, in 0.1 increments, respectively. A total of 100 pie charts was used. In the single-cue task, each pie chart was presented once in a random order. In the choice task, two pie charts were randomly allocated to the two options. During one session of electrophysiological recording, ∼30–60 trial blocks of the choice task were sometimes interleaved with 100–120 trial blocks of the single-cue task.

##### Calibration of the reward supply system

The precise amount of liquid reward was controlled and delivered to the monkeys using a solenoid valve. An 18 gauge tube (inner diameter, 0.9 mm) was attached to the tip of the delivery tube to reduce the variation across trials. The amount of reward in each payoff condition was calibrated by measuring the weight of water with 0.002 g precision (hence, 2 µl) on a single-trial basis. This calibration method was the same as previously used (Yamada et al., 2018).

#### Electrophysiological recordings

We used conventional techniques for recording the single-neuron activity from the DS, VS, cOFC, and mOFC. Monkeys were implanted with recording chambers (28 × 32 mm) targeting the OFC and striatum, centered 28 mm anterior to the stereotaxic coordinates. The locations of the chambers were verified using anatomic magnetic resonance imaging. At the beginning of recording sessions in a day, a stainless steel guide tube was placed within a 1 mm spacing grid, and a tungsten microelectrode (1–3 MΩ; FHC) was passed through the guide tube. To record neurons in the mOFC and cOFC, the electrode was lowered until it approximated the bottom of the brain after passing through the cingulate cortex and dorsolateral prefrontal cortex, or between them. For neuronal recording in the DS, the electrode was lowered until low spontaneous activity was observed after passing through the cortex and white matter. For recording in the VS, the electrode was lowered further until it passed through the internal capsule. At the end of VS recording sessions in a day, the electrode was occasionally lowered close to the bottom of the brain to confirm recording depth relative to the bottom. Electrophysiological signals were amplified, bandpass filtered, and monitored. Single-neuron activity was isolated based on spike waveforms. We recorded from the four brain regions of a single hemisphere of each of the two monkeys: 194 DS neurons (monkey SUN, 98; monkey FU, 96), 144 VS neurons (monkey SUN, 89; and monkey FU, 55), 190 cOFC neurons (monkey SUN, 98; monkey FU, 92), and 158 mOFC neurons (monkey SUN, 64; monkey FU, 94). The activity of all single neurons was sampled when the activity of an isolated neuron demonstrated a good signal-to-noise ratio (>2.5). Blinding was not performed. The sample sizes required to detect effect sizes (number of recorded neurons, number of recorded trials in a single neuron, and number of monkeys) were estimated in reference to previous studies (Yamada et al., 2013b, 2018; Chen and Stuphorn, 2015). Neural activity was recorded during 100–120 trials of the single-cue task. During choice trials, neural activity was not recorded. Presumed projection neurons (phasically active neurons; Yamada et al., 2016) were recorded from the DS and VS, while presumed cholinergic interneurons (tonically active neurons; Yamada et al., 2004; Inokawa et al., 2020) were not recorded.

### Statistical analysis

For statistical analysis, we used the statistical software package R (http://www.r-project.org/). All statistical tests for behavioral and neural analyses were two tailed.

In the present study, we used two variables for analyses: probability and magnitude. We defined the probability of reward from 0.1 to 1.0, and the magnitude of reward from 0.1 to 1.0 ml. Under this definition of units, the effects of probability and magnitude on the data were equivalent. Thus, data were not standardized in the analyses.

### Behavioral analysis

We examined whether the monkey's choice behavior depended on the expected values of the two options located on the left and right sides of the center. We pooled choice data across all recording sessions (monkey SUN: 884 sessions, 242 d; monkey FU: 571 sessions, 127 d), yielding 44,883 and 19,292 choice trials for monkeys SUN and FU, respectively. A percentage of the right target choices was estimated in the pooled choice data for all combinations of expected values of the left and right target options. The percentage of right target choices was also estimated in each recording session by segmenting the choice data as a function of the following seven conditions of difference in the expected values (right minus left) as follows: −1.0 to −0.5, −0.5 to −0.3, −0.3 to −0.1, −0.1 to 0.1, 0.1 to 0.3, 0.3 to 0.5, and 0.5 to 1.0. Reaction times to choose target options after the appearance of target options were estimated and analyzed with the expected value differences (right minus left) as follows: −1.0 to −0.5, −0.5 to −0.3, −0.3 to −0.1, −0.1 to 0.1, 0.1 to 0.3, 0.3 to 0.5, and 0.5 to 1.0.

#### Model fitting

The percentage of choosing the right-side option was analyzed in the pooled data using a general linear model with binominal distribution:

(1) $$P{\text{chooses}}_{R}=1/\left(1+e^{-z}\right),$$

where the relationship between *P*chooses*_R_* and *Z* was given by the logistic function in each of the following three models: number of pie segments (M1), probability and magnitude (M2), and expected values (M3).

The first model, M1, assumed that the monkeys chose a target by comparing the number of pie segments for two targets, as follows:

(2) $$Z=b_{0}+b_{1}N{\text{pie}}_{L}+b_{2}N{\text{pie}}_{R},$$

where *b*_0_ is the intercept and *N*pie*_L_* and *N*pie*_R_* are the number of pie segments contained in the left and right pie chart stimuli, respectively. Values of *b*_0_ to *b*_2_ were free parameters and estimated by maximizing the log likelihood.

The second model, M2, assumed that the monkeys chose a target by comparing the probability and magnitude of two targets, as follows:

(3) $$Z=b_{0}+b_{1}P_{L}+b_{2}P_{R}+b_{3}M_{L}+b_{4}M_{R},$$

where *b*_0_ is the intercept; *P_L_* and *P_R_* are the probability of rewards for left and right pie chart stimuli, respectively; and *M_L_* and *M_R_* are the magnitude of rewards for left and right pie chart stimuli, respectively. Values of *b*_0_ to *b*_4_ were free parameters and were estimated by maximizing the log likelihood.

The third model, M3, assumed that the monkeys chose a target by comparing the expected values of rewards for two targets, as follows:

(4) $$Z=b_{0}+b_{1}{\text{EV}}_{L}+b_{2}{\text{EV}}_{R},$$

where *b*_0_ is the intercept and EV*_L_* and EV*_R_* are the expected values of rewards as probability times magnitude for left and right pie chart stimuli, respectively. Values of *b*_0_ to *b*_2_ were free parameters and were estimated by maximizing the log likelihood.

#### Model comparisons

To identify the best structural model to describe the behavior of the monkeys, we compared the three models described above. In each model, we estimated a combination of best-fit parameters to explain the choice behavior of the monkeys. We compared their goodness-of-fit based on Akaike information criterion (AIC) and Bayesian information criterion (BIC; Burnham and Anderson, 2004),

(5) AIC (Model)=−2L+2k

(6) BIC (Model)=−2L+klogn,

where *L* is the maximum log-likelihood of the model, *k* is the number of free parameters, and *n* is the sample size. After estimating the best-fit parameters in each model, we selected one model that exhibited the smallest AIC and BIC. To evaluate model fits, we estimated a McFadden's pseudo-*r*^2^ statistic using the following equation:

(7) $$\text{Pseudo }r2=\left(L_{0}-L_{\text{Model}}\right)/L_{0},$$

where *L*_Model_ is the maximum log likelihood for the model given the data, and *L*_0_ is the log likelihood under the assumption that all free parameters are 0 in the model.

### Neural analysis

#### Basic firing properties

Peristimulus time histograms were drawn for each single-neuron activity aligned at visual cue onset. To display a color map histogram, a peak activity (maximum firing rate in each histogram) was detected for each neuron. The average activity curves were smoothed using a 50 ms Gaussian kernel (σ = 50 ms) and normalized by the peak firing rates. A percentage of neurons showing the activity peak during cue presentation was compared among the four brain regions using a χ^2^ test at *p* < 0.05. Peak firing rates, peak latency, and duration of peak activity (half-peak width) were compared among the four brain regions using parametric or nonparametric tests, with a statistical significance level of *p* < 0.05. Baseline firing rates during 1 s before the appearance of central fixation targets were also compared with a statistical significance level of *p* < 0.05.

#### Estimation of neural firing rates through task trials

We analyzed neural activity during a 2.7 s time period from the onset of pie chart stimuli to the onset of outcome feedback during the single-cue task. To obtain a time series of neural firing rates through a trial, we estimated the firing rates of each neuron for every 0.1, 0.05, or 0.02 s time window (without overlap) during the 2.7 s period. No Gaussian kernel was used.

#### Estimation of neural firing rates in a fixed time window

We analyzed neural activity during a 1 s time window after the onset of pie chart stimuli during the single-cue task. The 1 s activity was used for the conventional analyses below. No Gaussian kernel was used.

### Conventional analyses to detect neural modulations in each individual neuron

#### Linear regression and model selection

For conventional and standard analyses of neural modulations by the probability and magnitude indicated by pie chart stimuli, we used linear regression and model selection analyses. As above, we estimated the firing rate of each neuron during the 1 s period after the onset of pie chart stimulus during the single-cue task. No Gaussian kernel was used.

#### Linear regression

Neural discharge rates (*F*) were fitted by a linear combination of the following variables:

(8) $$F=b_{0}+b_{p}\text{ Probability}+b_{m}\text{ Magnitude,}$$

where Probability and Magnitude are the probability and magnitude of rewards indicated by the pie chart, respectively. *b*_0_ is the intercept. If *b_p_* and *b_m_* were not 0 at *p* < 0.05, discharge rates were regarded as being significantly modulated by that variable.

On the basis of the linear regression, activity modulation patterns were categorized into the following several types: “Probability” type with a significant *b_p_* and without a significant *b_m_*; “Magnitude” type without a significant *b_p_* and with a significant *b_m_*; “Expected value” type with significant *b_p_* and *b_m_* with the same sign (i.e., positive *b_p_* and positive *b_m_* or negative *b_p_* and negative *b_m_*); “Risk-Return” type with significant *b_p_* and *b_m_* with both having opposite signs (i.e., negative *b_p_* and positive *b_m_* or positive *b_p_* and negative *b_m_*); and “Nonmodulated” type without significant *b_p_* and *b_m_*. The Risk-Return types reflect high-risk high return (prefer low probability and large magnitude) or low-risk low return (prefer high probability and low magnitude).

#### Model selection

Neural discharge rates, *F*, were fitted using the following five models:

(9) $$\text{M}1:F=b_{\text{0}}$$

(10) $$\text{M}2:F=b_{\text{0}}+b_{p}\text{ Probability}$$

(11) $$\text{M}3:F=b_{\text{0}}+b_{m}\text{ Magnitude}$$

(12) $$\text{M}4:F=b_{\text{0}}+b_{p}\text{ Probability}+b_{m}\text{ Magnitude}$$

(13) $$\text{M}5:F=b_{\text{0}}+b_{\text{ev}}\text{ Expected value,}$$

where Expected value is the expected value estimated from the visual pie chart as probability multiplied by magnitude. *b*_0_ is the intercept. Probability and Magnitude are the probability and magnitude of reward indicated by the pie chart, respectively. Among the five models, we selected one model that exhibited the smallest AIC or BIC.

If the selected model was M1, neurons were defined as the Nonmodulated type. If the selected model was M2, neurons were defined as the Probability type. If the selected model was M3, neurons were defined as the Magnitude type. If the selected model was M4 with the same signs of *b_p_* and *b_m_*, neurons were defined as the Expected value type. If the selected model was M4 with opposite signs of *b_p_* and *b_m_*, neurons were defined as the Risk-Return type. If the selected model was M5, neurons were defined as the Expected value type.

#### Conventional analyses through task trials

We applied the three conventional analyses above (linear regression, AIC-based model selection, and BIC-based model selection) for the activity of neurons estimated at every time window in the four brain regions. As above, we estimated the firing rate of each neuron for every 0.1, 0.05, or 0.02 s time window (without overlap) during the 2.7 s period. No Gaussian kernel was used. The activity modulation type was defined in each time window during the 2.7 s period. The analyses described percentages of neural modulation types throughout cue presentation.

### Population dynamics using principal component analysis

#### Estimation of neuron firing rates through task trials

As above, we estimated the firing rate in each neuron for every 0.1, 0.05, or 0.02 s time window (without overlap) during the 2.7 s period. No Gaussian kernel was used.

#### Regression subspace

We used linear regression to determine how the probability and magnitude of rewards affect the activities of each neuron in the four neural populations. Each neural population was composed of all recorded neurons in each brain region. We first set the probability and magnitude as 0.1–1.0 and 0.1–1.0 ml, respectively. We then described the average firing rates of neuron *i* at time *t* as a linear combination of the probability and magnitude in each neural population, as follows:

(14) $${\text{F}}_{\left(i,t,k\right)}={\text{b}}_{\text{0}(\text{i,t})}+{\text{b}}_{1\text{(i,t)}}\mathrm{Probabilit}y_{\left(\text{k}\right)}+{\text{b}}_{2(i,t)}\mathrm{Magnitud}e_{\left(\text{k}\right)},$$

where *F*_(_*_i,t,k_*_)_ is the average firing rate of neuron *i* at time *t* on trial *k*, Probability_(_*_k_*_)_ is the probability of reward cued to the monkey on trial *k*, and Magnitude_(_*_k_*_)_ is the magnitude of reward cued to the monkey on trial *k*. The regression coefficients *b*_0(_*_i_*_,_*_t_*_)_ to *b*_2(_*_i_*_,_*_t_*_)_ describe the degree to which the firing rates of neuron *i* depend on the mean firing rates (hence, firing rates independent of task variables), the probability of rewards, and the magnitude of rewards, respectively, at a given time *t* during the trials.

We used the regression coefficients described in Equation 14 to identify how the dimensions of neural population signals were composed from the probability and magnitude as aggregated properties of individual neural activity. This step corresponds to the fundamental conceptual step of viewing the regression coefficients as a temporal structure of neural modulation by probability and magnitude at the population level. Our procedures are analogous to the state space analysis performed by Mante et al. (2013), in which the regression coefficients were used to provide an axis (or dimension) of the variables of interest in multidimensional state space obtained by principal component analysis (PCA). In the present study, our orthogonalized task design allowed us to reliably project neural firing rates into the regression subspace. Note that our analyses were not aimed at describing the population dynamics of neural signals as a trajectory in the multidimensional task space, which is the standard goal of state space analysis.

#### Principal component analysis

We used PCA to identify dimensions of the neural population signal in the orthogonal spaces composed of the probability and magnitude of rewards in each of the four neural populations. In each neural population, we first prepared a two-dimensional data matrix *X* of size *N*_(neuron)_ × *N*_(_*_C_*_×_*_T_*_)_; the regression coefficient vectors *b*_1(_*_i_*_,_*_t_*_)_ and *b*_2(_*_i,t_*_)_ in Equation 14, whose rows correspond to the total number of neurons in each neural population and columns correspond to *C*, the total number of conditions (i.e., two: probability and magnitude), and T is the total number of analysis windows (i.e., 2.7 s divided by the window size). A series of eigenvectors was obtained by applying PCA once to the data matrix *X* in each of the four neural populations. The principal components (PCs) of this data matrix are vectors *v*_(_*_a_*_)_ of length *N*_(neuron)_, the total number of recorded neurons if *N*_(_*_C_*_×_*_T_*_)_ is > *N*_(neuron)_; otherwise, the length is *N*_(_*_C_*_×_*_T_*_)_. PCs were indexed from the principal components, explaining the most variance to the least variance. The eigenvectors were obtained using the prcomp function in R software. It must be noted that we did not perform denoising in the PCA (Mante et al., 2013), since we did not aim to project firing rates into state space. Instead, we intended to use the PCs to identify the main features of neural modulation signals at the population level through task trials.

#### Eigenvectors

When we applied PCA to the data matrix *X*, we could deconstruct the matrix into eigenvectors and eigenvalues. The eigenvectors and eigenvalues exist as pairs with every eigenvector having a corresponding eigenvalue. In our analysis, the eigenvectors at time *t* represent a vector in the space of probability and magnitude. The eigenvalues at time *t* for the probability and magnitude were scalars, indicating the extent of variance in the data in that vector. Thus, the first PC is the eigenvector with the highest eigenvalue. We mainly analyzed eigenvectors for the first PC (PC1) and PC2 in the following analyses. Note that we applied PCA once to each neural population, and, thus, the total variances contained in the data were different among the four populations.

#### Analysis of eigenvectors

We evaluated the characteristics of eigenvectors for PC1 and PC2 in each of the four neural populations in terms of the vector angle, size, and deviation in the space of probability and magnitude. The angle is the vector angle from the horizontal axis from 0° to 360°. The size is the length of the eigenvector. The deviation is the difference between vectors. We estimated the deviation from the mean vector in each neural population. These three characteristics of the eigenvectors were compared among the four neural populations at *p* < 0.05, using the Kruskal–Wallis test and the Wilcoxon rank-sum test with Bonferroni correction for multiple comparisons. The vector during the first 0.1 s was extracted from these analyses.

#### Shuffle control for PCA

To examine the significance of population structures described by PCA, we performed two shuffle controls. When we projected the neural activity into the regression subspace, data were randomized by shuffling in two ways. In shuffled condition 1, *b*_1(_*_i,t_*_)_ and *b*_2(_*_i,t_*_)_ in Equation 14 were estimated with the randomly shuffled allocation of trial number *k* to the Probability_(_*_k_*_)_ and Magnitude_(_*_k_*_)_ only once for all time *t* in each neuron. This shuffle provided a data matrix *X* of size *N*_(neuron)_ × *N*_(_*_C_*_×_*_T_*_)_, eliminating the modulation of probability and magnitude observed in condition *C*, but retaining the temporal structure of these modulations across time. In shuffled condition 2, *b*_1(_*_i,t_*_)_ and *b*_2(_*_i,t_*_)_ in Equation 14 were estimated with the randomly shuffled allocation of trial number *k* to the Probability_(_*_k_*_)_ and Magnitude_(_*_k_*_)_ at each time *t* in each neuron. This shuffle provided a data matrix *X* of size *N*_(neuron)_ × *N*_(_*_C_*_×_*_T_*_)_, eliminating the structure across conditions and times. In these two shuffle controls, matrix *X* was estimated 1000 times. PCA performance was evaluated by constructing distributions of the explained variances for PC1 to PC4. The statistical significance of the variances explained by PC1 and PC2 was estimated based on bootstrap SEs (i.e., SD of the reconstructed distribution).

#### Bootstrap resampling for onset and peak latencies

To detect the onset and peak latencies of population signals, we analyzed dynamic changes in the population structure with the size of eigenvector in each neural population. We used a time series of eigenvectors in 0.02 s analysis windows and estimated the sizes of the time series of vectors for PC1. To obtain smooth changes in the vector size, a cubic spline function was applied with a resolution of 0.005 s. Vector sizes during a 0.3 s baseline period were obtained by applying PCA to the matrix data *X* with time *t* from 0.3 s before cue onset to the onset of feedback (i.e., 3.0 s time period). An SD of vector sizes during the 0.3 s baseline period before cue onset was obtained for each neural population. The onset latency of the population signal was defined as the time when the spline curve was >3 SDs during the baseline period. The peak latency of the population signal was defined as the time from cue onset to the time when the maximum vector size was obtained.

We estimated mean latencies of the onset and peak using a parametric bootstrap resampling method (Efron and Tibshirani, 1993). In each neural population, the neurons were randomly resampled with a duplicate, and a data matrix *X* of size *N*_(neuron)_ × *N*_(_*_C_*_×_*_T_*_)_ was obtained. The PCA was applied to the data matrix *X.* The time series of eigenvectors was obtained, and their sizes were estimated. The onset and peak latencies were estimated as above. This resampling was conducted 1000 times, and distributions of the onset and peak latencies were obtained. The statistical significance of the onset and peak latencies was estimated based on the bootstrap SEs (i.e., SD of the reconstructed distribution).

#### Neural population structure with expected value subspace

To include the expected value (i.e., multiplicative integration) directly into the state space analysis, we used the following regression model, which described the average firing rates *F*_(_*_i,t,k_*_)_ of neuron *i* at time *t* as the expected value on trial *k* in each neural population, as follows:

(15) $${\text{F}}_{\left(i,t,k\right)}={\text{b}}_{0(i,t)}+{\text{b}}_{3(i,t)}\mathrm{Expected}{\mathrm{value}}_{\left(\text{k}\right)}.$$

We prepared a two-dimensional data matrix *X* of size *N*_(neuron)_ × *N*_(_*_C_*_×_*_T_*_)_ under three conditions (probability, magnitude, and expected value); the regression coefficient vectors *b*_1(_*_i,t_*_)_ and *b*_2(_*_i,t_*_)_, in Equation 14, and *b*_3(_*_i,t_*_)_ in Equation 15. We applied PCA to the data matrix *X* in each neural population. Note that Equation 15 explains some of the same variances as the neural modulation defined in Equation 14 for each neuron, but separately used from Equation 14 to project neural activity into the expected value subspace.

## Results

### Task and behavior in monkeys

**Figure 1.**
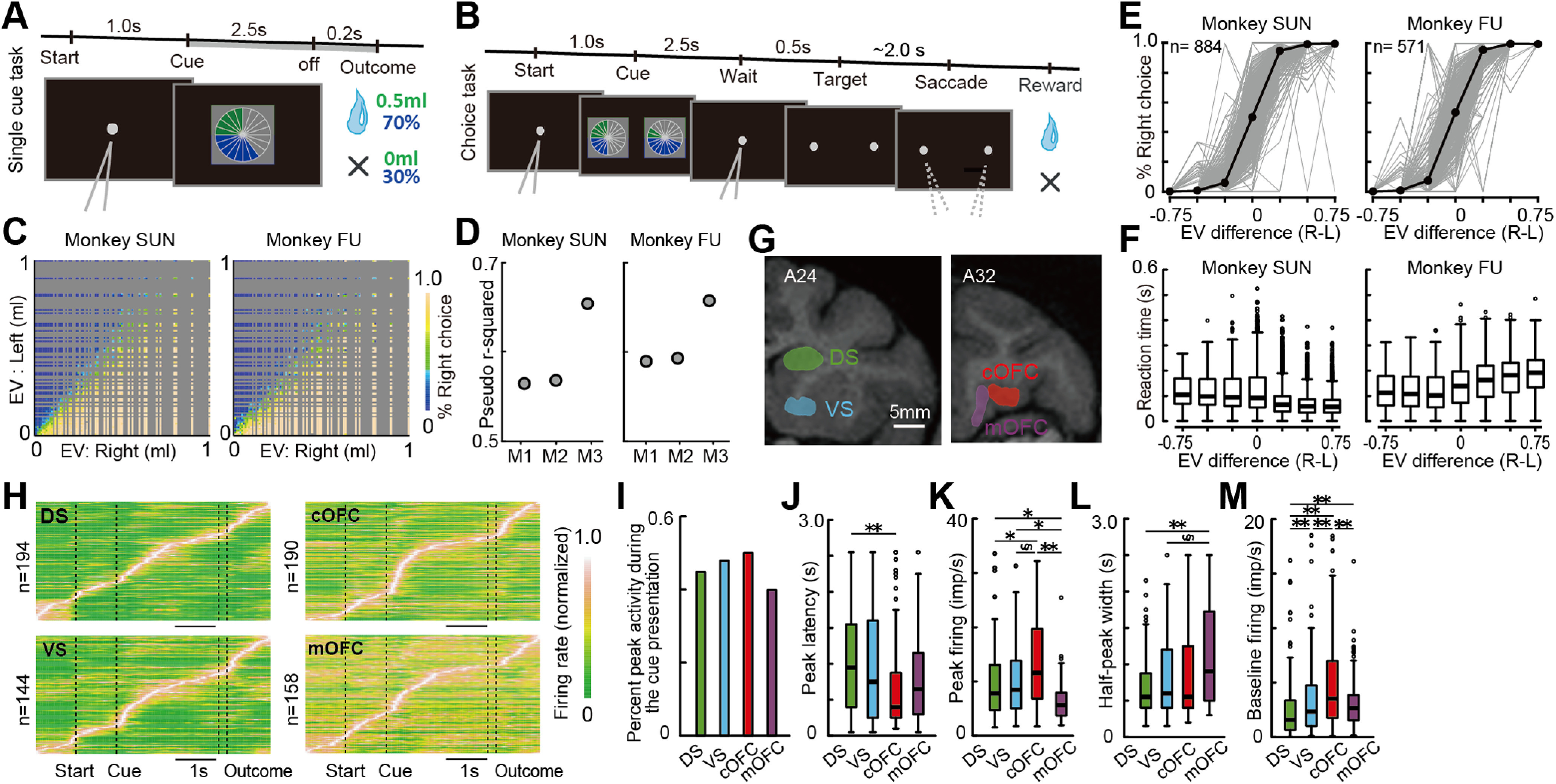
Task, behavior, and basic firing properties of neurons. ***A***, Sequence of events during the single-cue task. A single visual pie chart having green and blue pie segments was presented to the monkeys. ***B***, Choice task. Two visually displayed pie charts were presented to the monkeys on the left and right sides of the center. After visual fixation of the reappeared in the central area of the target, the central fixation target disappeared, and monkeys chose either of the targets by fixating on it. A block of the choice trials was sometimes interleaved between the single-cue trial blocks. During the choice trials, neural activity was not recorded. ***C***, Percentages of right target choice during the choice task plotted against the EVs of the left and right target options. Aggregated choice data were used. ***D***, Pseudo-*r*^2^ estimated in the three behavioral models: M1, number of pie segments; M2, probability and magnitude; M3: expected values. ***E***, Percentage of right target choices estimated in each recording session (gray lines) plotted against the difference in expected values (right minus left). The choice data were segmented by seven conditions of the difference in the expected values, as follows: −1.0 to −0.5, −0.5 to −0.3, −0.3 to −0.1, −0.1 to 0.1, 0.1 to 0.3, 0.3 to 0.5, and 0.5 to 1.0. Black plots indicate the mean. ***F***, Reaction time to choose a target option plotted against the difference in expected values (right minus left) as −1.0 to −0.5, −0.5 to −0.3, −0.3 to −0.1, −0.1 to 0.1, 0.1 to 0.3, 0.3 to 0.5, and 0.5 to 1.0. ***G***, An illustration of neural recording areas based on sagittal MR images. Neurons were recorded from the mOFC (14O, orbital part of area 14) and cOFC (area 13 M) at the A31–A34 anterior–posterior (A–P) level. Neurons were also recorded from the DS and VS, respectively, at the A21–A27 level. White scale bar, 5 mm. ***H***, Color map histograms of neuronal activities recorded from the four brain regions. Each horizontal line indicates neural activity aligned to cue onset averaged for all lottery conditions. Neuronal firing rates were normalized to the peak activity. ***I***, Percentages of neurons showing an activity peak during cue presentation. ***J***, Box plots of peak activity latency after cue presentation. ***K***, Firing rates of peak activity observed during cue presentation. ***L***, Box plots of half-peak width, indicating the phasic nature of activity changes. ***M***, Box plots of baseline firing rates during the 1 s time period before the onset of the central fixation target. In ***J–M***, asterisks indicate statistical significance among two neural populations using the Wilcoxon rank-sum test with Bonferroni correction for multiple comparisons [statistical significance: **p < 0.01, *p < 0.05, and §0.05 < *p* < 0.06 (close to significance), respectively].

Based on the vast literature on human behavioral economics and by harnessing the well developed visual and cognitive abilities in nonhuman primates, we designed a behavioral task in which monkeys estimated the expected values of rewards from numerical symbols, mimicking events performed by humans. The task involved a visual pie chart that included two numerical symbols associated with the probability and magnitude of fluid rewards with great precision. After monkeys fixated a central gray target, a visual pie chart comprising green and blue pie segments was presented (Fig. 1*A*). The number of green pie segments indicated the magnitude of fluid rewards in 0.1 ml increments (0.1–1.0 ml). Simultaneously, the number of blue pie segments indicated the probability of reward in 0.1 increments (0.1–1.0 where 1.0 indicates a 100% chance). After a 2.5 s delay, the visual pie chart disappeared, and a reward outcome was provided to the monkeys with the indicated amount and probability of reward, unless no reward was given. Under this experimental condition, the expected values of rewards are defined as the probability multiplied by the magnitude cued by the numerical symbols.

To examine whether the monkeys accurately perceived the expected values from the numerical symbols for probability and magnitude, we applied a choice task to the monkeys (Fig. 1*B*). Analysis of the aggregated choice data indicated that the two monkeys exhibited nearly efficient performance in selecting a larger expected value option among two alternatives during choice trials (Fig. 1*C*). We examined which of the following three behavioral models best described the behavior of the monkey, as follows: M1, monkeys make choices based on the number of pie segments; M2, monkeys make choices based on the probability and magnitude; and M3, monkeys make choices based on the expected value. Comparisons of the model performances based on AIC and BIC (Burnham and Anderson, 2004) revealed that model three best explained the behavior of the monkey, as indicated by the smallest AIC and BIC values (monkey SUN: AIC: M1, 27,105; M2, 26,895; M3, 21,539; BIC: M1, 27,131; M2, 26,939; M3, 21,565; monkey FU: AIC: M1, 10,980; M2, 10,889; M3, 9166; BIC: M1, 11,003; M2, 10,929; M3, 9190). Model three consistently showed the highest pseudo-*r*^2^ values in each monkey (Fig. 1*D*). These results indicate that monkeys used the expected values estimated from the numerical symbols for probability and magnitude.

We also evaluated the choice behaviors of the monkey by analyzing the percentage of choices among two lottery options session by session. Each monkey showed a certain variance in the percent choices over sessions (Fig. 1*E*, gray), although choices in each monkey were clearly dependent on the expected value difference between the two options, without a clear choice-side bias on average (Fig. 1*E*, black). In contrast, reaction times to choose the target option showed a choice-side bias without a consistent dependence on the expected value differences between the two monkeys (Fig. 1*F*). Monkey SUN showed longer reaction times when the expected values of the left-side options were larger than those of right-side options, while monkey FU showed longer reaction times when the expected values of the right-side options were larger (Kruskal–Wallis test; monkey SUN: *n* = 44,883, *p* < 0.001, *H* = 4000, df = 6; monkey FU: *n* = 19,292, *p* < 0.001, *H* = 1710, df = 6). These results indicate that the behavior of the monkeys depended to a certain extent on the expected value difference.

### Neural population data

We constructed four pseudo-populations of neurons by recording single-neuron activity during the single-cue task (Fig. 1*A*) from the DS (194 neurons), VS (144 neurons), cOFC (190 neurons), and mOFC (158 neurons; Fig. 1*G*). The four constructed neural populations exhibited changes in their activities at different times in the task trials (Fig. 1*H*). Approximately 40–50% of neurons in the four neural populations demonstrated peak activity during cue presentation (Fig. 1*I*; χ^2^ test: *n* = 686, *p* = 0.32, χ^2^ = 3.55, df = 3), with several basic firing properties (Fig. 1*J–M*). Strong peak activities with short latency were observed in the cOFC (Kruskal–Wallis test; latency: Fig. 1*J*, *n* = 314, *p* = 0.013, *H* = 10.9, df = 3; peak firing rate: Fig. 1*K*, *n* = 314, *p* < 0.001, *H* = 32.1, df = 3). Activity changes were slow in the mOFC (Fig. 1*L*; Kruskal–Wallis test; *n* = 314, *p* = 0.003, *H* = 13.4, df = 3). Baseline firing rates were the highest in the cOFC (Fig. 1*M*; Kruskal–Wallis test; *n* = 686, *p* < 0.001, *H* = 60.3, df = 3). In short, strong activity with short latency frequently occurred in the cOFC in contrast to the phasic activity at various latencies in the DS and VS and the relatively tonic and gradual activity changes in the mOFC.

### Conventional analyses for detecting expected value signals

**Figure 2.**
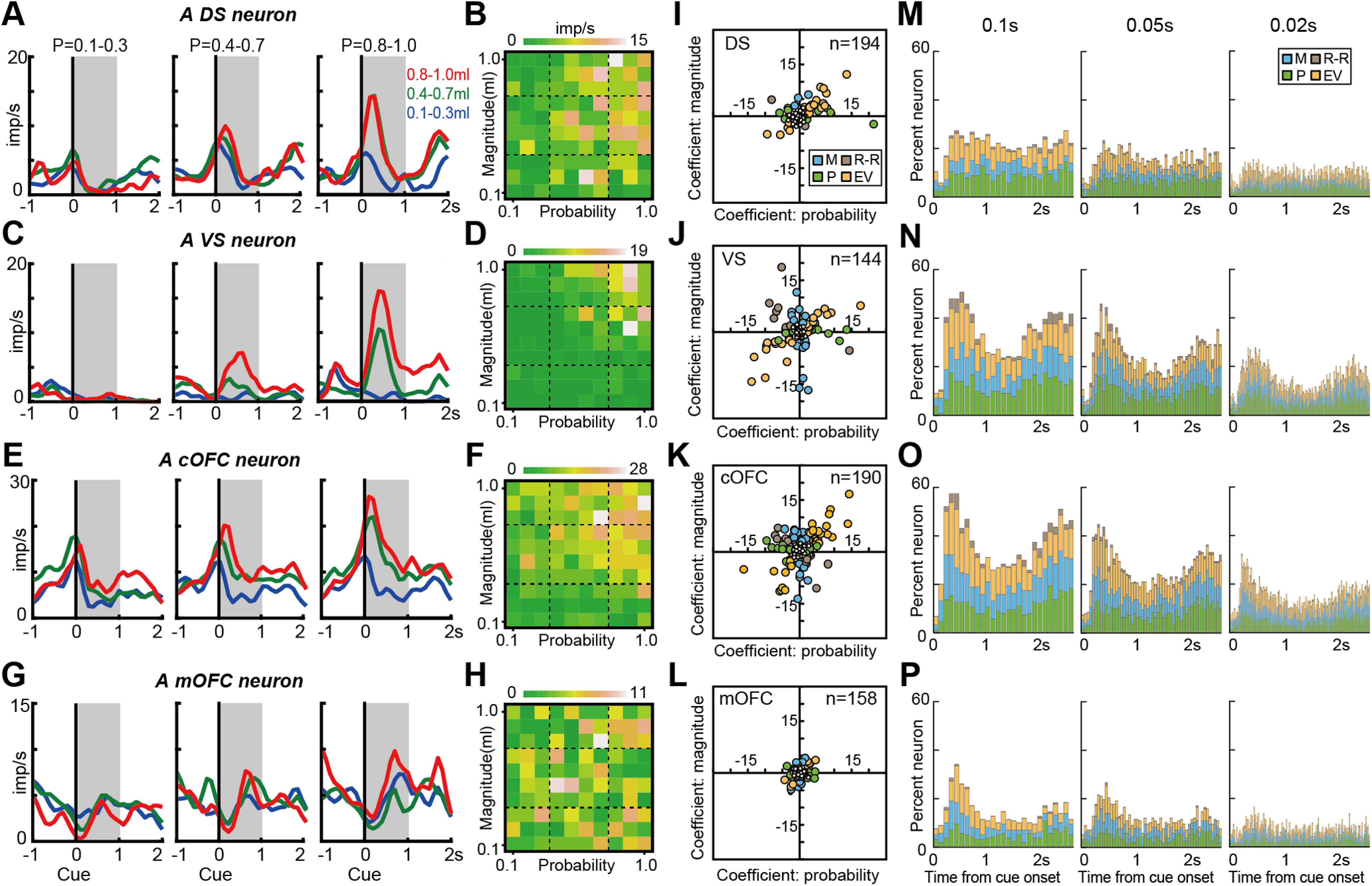
Expected value signals detected by conventional analyses. ***A***, Example activity histogram of a DS neuron modulated by expected value during the single-cue task. The activity aligned to the cue onset is represented for three different levels of probability (0.1–0.3, 0.4–0.7, and 0.8–1.0) and magnitude (0.1–0.3, 0.4–0.7, and 0.8–1.0 ml) of rewards. Gray hatched time windows indicate the 1 s time window used to estimate the neural firing rates shown in ***B***. The neural modulation pattern was defined as the expected value type based on all three analyses (linear regression, AIC-based model selection, and BIC-based model selection). Regression coefficients for probability and magnitude were 6.17 (*p* < 0.001) and 2.54 (*p* = 0.007), respectively. ***B***, An activity plot of the DS neuron during the 1 s time window shown in ***A*** against the probability and magnitude of rewards. ***C***, ***D***, Same as ***A*** and ***B***, but for a VS neuron defined as the expected value type based on all three analyses. Regression coefficients for probability and magnitude were 7.14 (*p* < 0.001) and 6.71 (*p* < 0.001), respectively. ***E***, ***F***, Same as ***A*** and ***B***, but for a cOFC neuron defined as the expected value type based on all three analyses. Regression coefficients for probability and magnitude were 8.55 (*p* < 0.001) and 11.1 (*p* < 0.001), respectively. ***G***, ***H***, Same as ***A*** and ***B***, but for an mOFC neuron. The neural modulation pattern was defined as the expected value type based on the AIC-based model selection, as the probability type based on the linear regression, and as the nonmodulated type based on the BIC-based model selection. Regression coefficients for probability and magnitude were 1.76 (*p* = 0.032) and 0.50 (*p* = 0.54), respectively. ***I–L***, Plots of regression coefficients for the probability and magnitude of rewards estimated for all neurons in the DS (***I***), VS (***J***), cOFC (***K***), and mOFC (***L***). Filled colors indicate the neural modulation pattern classified by the BIC-based model selection. P, Probability type; M, magnitude type, EV: Expected value type, and R-R: Risk-Return type. The nonmodulated type is indicated by the small open circle. ***M–P***, Percentages of neural modulation types based on BIC-based model selection through cue presentation in the DS (***M***), VS (***N***), cOFC (***O***), and mOFC (***P***). The analysis window size is 0.1 s (left), 0.05 s (middle), and 0.02 s (right), respectively.

**Figure 3.**
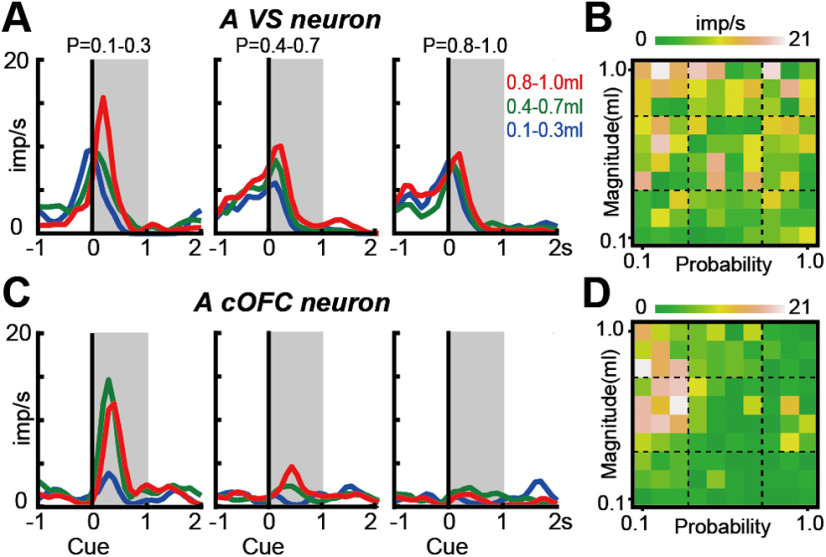
Risk-return signals detected by conventional analyses. ***A***, Example activity histogram of a VS neuron modulated by both probability and magnitude of rewards with opposite signs (i.e., negative *b_p_* and positive *b_m_*). The activity aligned to cue onset is represented for three different levels of probability (0.1–0.3, 0.4–0.7, and 0.8–1.0) and magnitude (0.1–0.3, 0.4–0.7, and 0.8–1.0 ml) of rewards. Gray hatched areas indicate a 1 s time window to estimate the neural firing rates shown in ***B***. The neural modulation pattern was defined as the risk–return type based on the linear regression and AIC-based model selection, and as the magnitude type based on the BIC-based model selection. Regression coefficients were −2.44 (*p* = 0.039) and 4.86 (*p* < 0.001) for probability and magnitude, respectively. ***B***, Activity plots of the VS neuron during the 1 s time window shown in ***A*** against the probability and magnitude of rewards. ***C***, ***D***, Same as ***A*** and ***B***, but for a cOFC neuron. The neural modulation type was defined as the risk–return type based on all three analyses. Regression coefficients for probability and magnitude were −6.65 (*p* < 0.001) and 3.82 (*p* < 0.001), respectively.

We first applied common conventional analyses (linear regression, AIC-based model selection, and BIC-based model selection) to the four neural populations to examine neural modulations by probability, magnitude, and expected value at a single-neuron level (see Materials and Methods). During a fixed 1 s time window after cue onset, these analyses showed that neurons in all four brain regions signal probability, magnitude, and expected value to some extent (Fig. 2). For example, neurons signaling expected value were found in each brain region (Fig. 2*A–H*). In addition, neurons signaling probability or magnitude were also observed in each brain region (Fig. 2*I–L*, blue, and green). Moreover, a subset of neurons in the cOFC and VS signaled high risk, high return or low risk, low return (Fig. 3). These neurons were characterized by a strong activity, which was elicited when the cue indicated low probability and large magnitude (hence, high risk, high return; Fig. 2*J*,*K*, brown). Indeed, each neural population was composed of a mixture of these signals (Fig. 2*I–L*), indicating that signals for the expected value and its components (i.e., probability and magnitude) appeared in each neural population during 1 s after cue onset. Note that the classification of neural modulation types was dependent on the analysis methods; however, the overall tendency for differences in neural modulations among neural populations was consistent among all three analyses.

We analyzed these neural modulation patterns through a task trial using these conventional analyses (Fig. 2*M–P*). We found no significant difference in the proportions of neural modulation types in the 0.1 s analysis window, except for the VS (χ^2^ test: DS: *n* = 104, df = 75, χ^2^ = 91.4, *p* = 0.096; VS: *n* = 104, df = 75, χ^2^ = 98.2, *p* = 0.037; cOFC: *n* = 104, df = 75, χ^2^ = 83.2, *p* = 0.242; mOFC: *n* = 104, df = 75, χ^2^ = 79.0, *p* = 0.353). Using a finer time resolution, a 10^−2^ s time resolution (0.02 s), the detected neural modulations were proportionally very small because signal-to-noise ratios generally decrease with the window size. These observations suggested that conventional analyses provided neural modulation patterns similar to those of previous studies, but they did not clearly provide evidence of temporal dynamics in the modulation patterns of neural populations. Thus, we developed an analytic tool to examine how the detection and integration of probability and magnitude are processed within these neural population ensembles.

### State space analysis for detecting neural population dynamics

**Figure 4.**
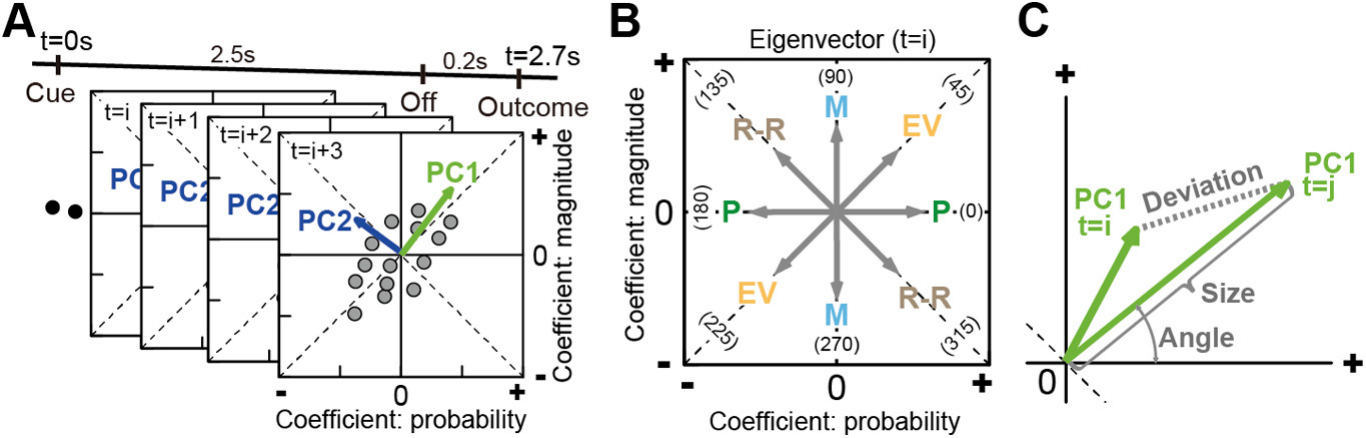
Schematic depictions for the analysis of neural population dynamics using PCA. ***A***, Time series of a neural population activity projected into a regression subspace composed of probability and magnitude. A series of eigenvectors was obtained by applying PCA once to each of the four neural populations. PC1 and PC2 indicate the first and second principal components, respectively. The number of eigenvectors obtained by PCA was 2.7 s divided by the analysis window size for the probability and magnitude: 27, 54, and 135 eigenvectors in a 0.1, 0.05, or 0.02 s time window, respectively. ***B***, Examples of eigenvectors at time of *i*th analysis window for probability and magnitude, whose direction indicates a signal characteristic at the time represented on the population ensemble activity. EV, 45°, 225°; M, magnitude (90°, 270°); P, probability (0°,180°); R-R, 135°, 315°. ***C***, Characteristics of the eigenvectors evaluated quantitatively. Angle, Vector angle from the horizontal axis taken from 0° to 360°. Size, Eigenvector length; deviation, difference between vectors.

State space analysis can provide temporal dynamics of neural population signal related to cognitive and motor performances (Churchland et al., 2012; Mante et al., 2013). In our lottery task, such population dynamics can describe how expected values evolved within neural population ensembles. To describe how each neural population detects and integrates probability and magnitude into the expected value, we represented each neural population signal as a vector time series in the space of probability and magnitude in two steps. First, we used linear regression to project a time series of each neural activity into a regression subspace composed of the probability and magnitude in each neural population. This step captures the across-trial variance caused by the probability and magnitude moment by moment at the population level. Second, we applied PCA to the time series of neural activities in the regression subspace in each neural population. This step determines the main feature of the neural population signal moment by moment in the space of probability and magnitude. Because activations are dynamic and change over time, the analysis identified whether and how signal transformations occurred to convert probability and magnitude into the expected value as a time series of eigenvectors (Fig. 4*A*). The directions of these eigenvectors capture the expected values as an angle moment by moment at the population level (Fig. 4*B*).

We evaluated eigenvector properties for PC1 and PC2 in each neural population in terms of vector angle, size, and deviation (Fig. 4*C*). A stable population signal is described as a small variation in eigenvector properties throughout a trial, whereas an unstable population signal is described as a large variation in eigenvector properties. It must be noted that our procedure is a variant of the state space analysis in line with the use of linear regression to identify dimensions of a neural population signal (Mante et al., 2013; Chen and Stuphorn, 2015); however, it was not aimed at projecting the population activity as trajectories in multidimensional space.

### Stable and unstable neural population signals

**Figure 5.**
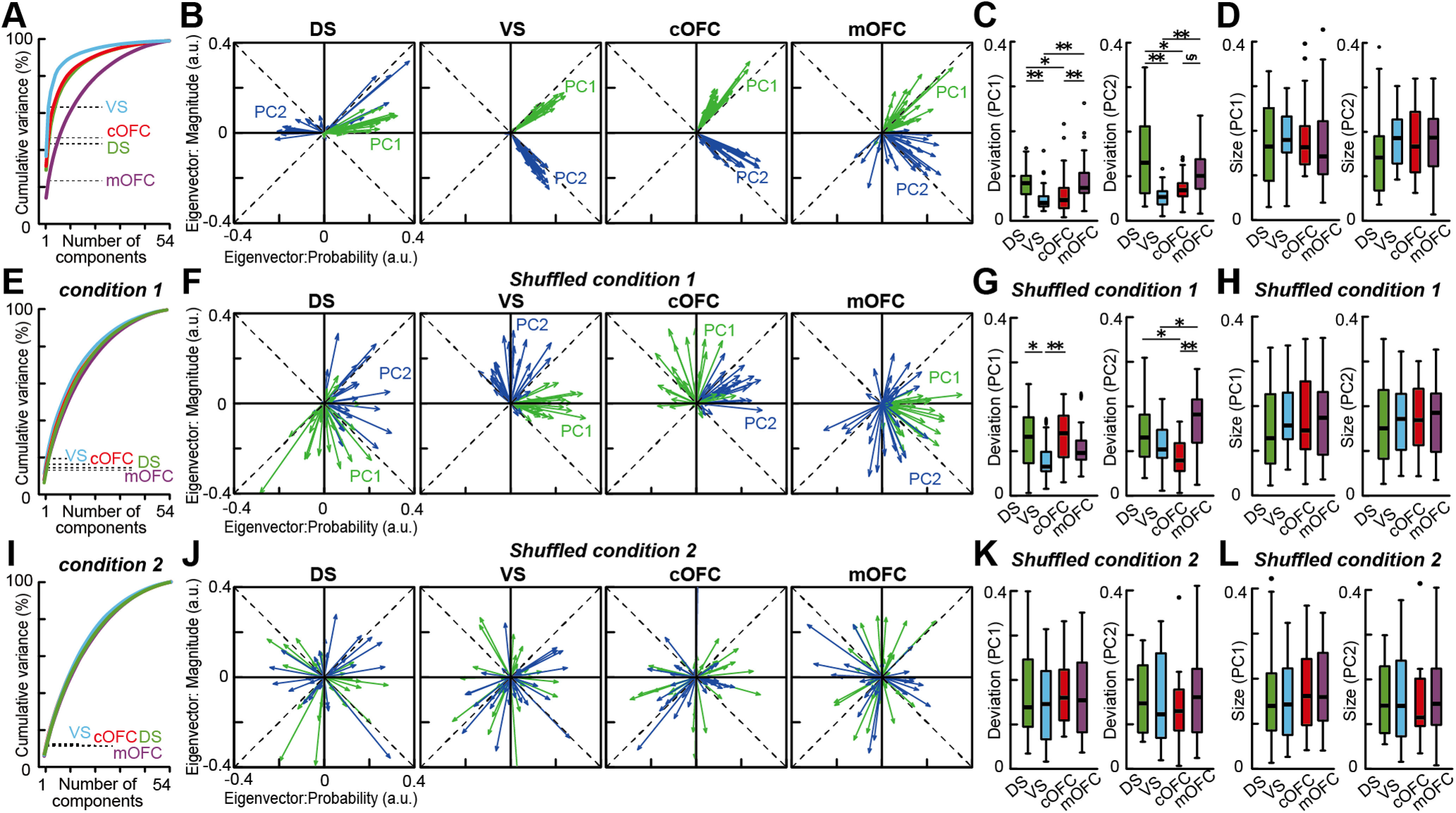
Neural populations provide stable expected value signals in the VS and cOFC. ***A***, Cumulative variance explained by PCA in the four neural populations. Dashed line indicates percentages of variances explained by PC1 and PC2 in each neural population. ***B***, Overlay plots of series of eigenvectors for PC1 and PC2 in the four neural populations. a.u., Arbitrary unit. ***C***, Box plots of vector deviation from the mean vector estimated in each neural population for PC1 (left) and PC2 (right). ***D***, Box plots of vector size estimated in each neural population for PC1 (left) and PC2 (right). ***E–H***, Same as ***A*–*D***, but for the PCA under the shuffled condition 1. See Materials and Methods for details. ***I–L***, Same as ***A–D***, but for the PCA under the shuffled condition 2. In ***C***, ***D***, ***G***, ***H***, ***K***, and ***L***, asterisks indicate statistical significance between two populations using the Wilcoxon rank-sum test with Bonferroni correction for multiple comparisons [statistical significance at **p < 0.01, *p < 0.05, and §0.05 < *p* < 0.06 (close to significance), respectively]. The results are shown by using a 0.1 s analysis window.

The eigenvector analyses yielded clear differences in neural population signals among the four populations (Fig. 5*A–D*). We first confirmed adequate performance of the state space analysis indicated by the percentages of variance explained in each population (Fig. 5*A*). The VS population exhibited the highest performance among the four neural populations, followed by the cOFC and DS populations, with the lowest performance exhibited by the mOFC population. Thus, the performance to process probability and magnitude information was distinct among the four neural populations.

To characterize the whole structure of each neural population signal, we analyzed the aggregated properties of the eigenvectors without their temporal order through a task trial. We first examined eigenvector properties for PC1. The aggregated eigenvectors revealed both stable and unstable neural population signals during cue presentation (Fig. 5*B*, green). The VS population exhibited the highest performance (37%) with eigenvectors for PC1 being stable throughout cue presentation, and directions close to 45°, that is, the expected value (Fig. 5*B*: VS, vector angle, PC1; mean ± SEM, 37.5° ± 0.98, 7.5° difference from 45°). The cOFC population also exhibited a stable expected value signal with the second-best performance (31%), but they deviated more from the ideal expected value signal (Fig. 5*B*: cOFC, vector angle, PC1; mean ± SEM, 59.4° ± 1.16, 14.4° difference from 45°; Wilcoxon rank-sum test, *n* = 52, *W* = 122, *p* < 0.001). Vector stability was the best in the VS and cOFC, as indicated by the smallest deviation from its mean vector among the four neural populations (Fig. 5*C*, left, PC1). Thus, VS and cOFC populations signaled expected values in a stable manner.

In contrast, unstable population signals were observed in the DS and mOFC (Fig. 5*B*, green). The DS population showed considerable variability in its eigenvectors (Fig. 5*C*, left, PC1) compared with those in the VS and cOFC neural populations. The signal carried by the DS neural population was close to 0°, that is, the probability (Fig. 5*B*: DS, vector angle, PC1; mean ± SEM, DS, 11.4° ± 1.72) with a performance closer to that of the cOFC (29%). The mOFC population exhibited a large variability in the eigenvectors (Fig. 5*B*: mOFC, PC1, vector angle; mean ± SEM, 38.1° ± 5.80; Fig. 5*C*, left, PC1) because of the poorest performance of PCA (14%), indicating a weak and fluctuating population signal. Thus, neural populations in the DS and mOFC did not signal expected value through cue presentation because of the dynamic changes and weakness of the signals, respectively.

Second, we examined eigenvector properties for PC2. The eigenvectors for PC2 revealed another feature of neural population signal, which reflected risk–return in the VS and cOFC (Fig. 5*B*, blue; vector angle, PC2; mean ± SEM, VS, 306.7° ± 1.07, 8.3° difference from 315°; cOFC, 322.4° ± 1.94, 7.4° difference from 315°). The deviations from the ideal risk–return signal were not significantly different between the VS and cOFC populations (Wilcoxon rank-sum test, *n* = 52, *W* = 319, *p* = 0.737). These signals were equally stable in the VS and cOFC (Fig. 5*C*, right, PC2). In clear contrast, DS and mOFC signals were unstable and fluctuated more (Fig. 5*C*, right, vector angle, PC2; mean ± SEM, DS, 64.8 ± 19.0; mOFC, 320.2 ± 8.77), similar to those observed for PC1 (Fig. 5*C*, left, PC1). Thus, the VS and cOFC were key brain regions to signal risk–return as well as expected value within their neural population ensembles, suggesting that integrated information of the probability and magnitude could be signaled in these neural populations.

**Figure 6.**
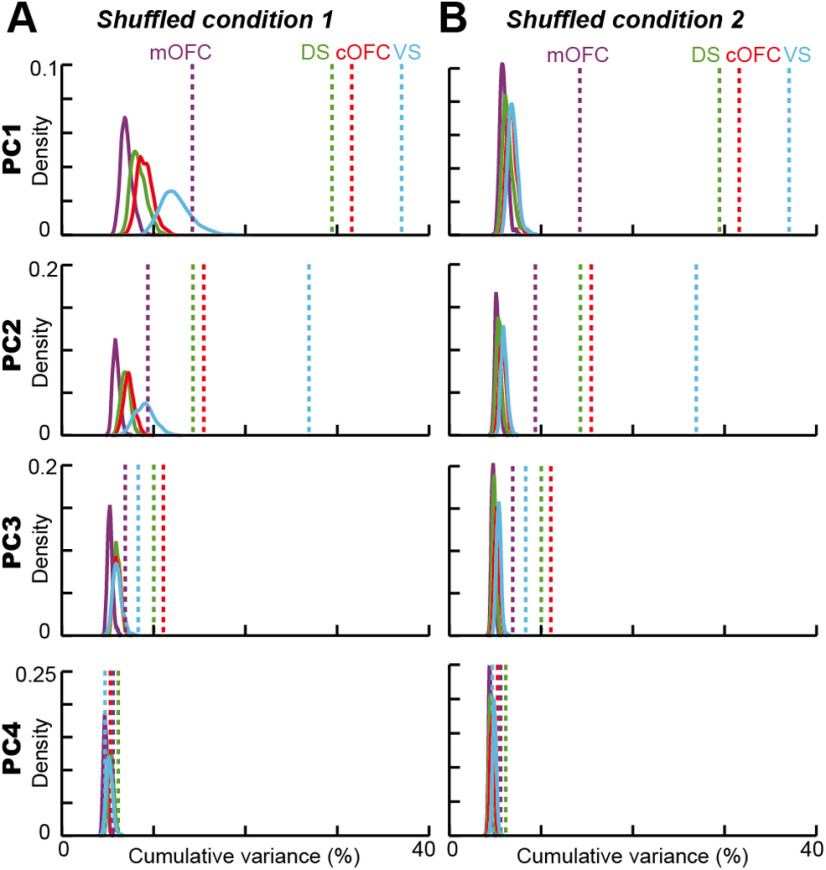
Probability density of explained variances by PCA in shuffled controls. ***A***, Probability density of variances explained by PCA for PC1 to PC4 under the shuffled condition 1 (for details, see Materials and Methods). The probability density was estimated with 1000 repeats of the shuffle in each neural population. ***B***, Probability density of variance explained by PCA for PC1 to PC4 under the shuffled condition 2 (for details, see Materials and Methods). The probability density was estimated with 1000 repeats of the shuffle in each neural population. In ***A*** and ***B***, dashed lines indicate the variances explained by PCA in each of the four neural populations without the shuffle. The results are shown by using 0.1 s analysis window.

To further examine the significance of these findings, we used a shuffle control procedure in two ways (see Materials and Methods). First, we randomly shuffled the allocation of probability and magnitude conditions to neural activity in each trial for each neuron (shuffled condition 1). When we shuffled the linear projection of neural activity into the regression subspace in this way, the neural population structure disappeared in all four brain regions (Fig. 5*F*). PCA performances for PC1 and PC2 were all <20% (Fig. 5*E*) and were significantly reduced from the observed data in all four brain regions, even in the mOFC (Fig. 6*A*; explained variance, *p* < 0.001 for all populations in PC1 and PC2). In addition, because of the shuffle, vector angles for PC1 and PC2 were changed compared with those from the original data (Fig. 5*B*,*F*). Eigenvector deviations under the shuffle control increased in most cases for PC1 (Fig. 5*G*; Wilcoxon rank-sum test, *n* = 52; PC1, DS, *W* = 237, *p* = 0.027; VS, *W* = 191, *p* = 0.002; cOFC, *W* = 132, *p* < 0.001; mOFC, *W* = 262, *p* = 0.078; PC2, DS, *W* = 352, *p* = 0.837; VS, *W* = 104, *p* < 0.001; cOFC, *W* = 331, *p* = 0.571; mOFC, *W* = 189, *p* = 0.002), with significant differences among the four neural populations (Fig. 5*G*; Kruskal–Wallis test; PC1, *n* = 104, df = 3, *H* = 16.4, *p* < 0.001; PC2, *n* = 104, df = 3, *H* = 21.4, *p* < 0.001). This might have occurred because the temporal structure of neural modulation was maintained through a trial in this shuffled condition 1.

We also tested another shuffle control, in which the trial conditions were shuffled in each analysis window throughout a trial (shuffled condition 2). Under this full-shuffle control, PCA performances decreased further, albeit slightly (Figs. 5*I*, 6*B*), without significant differences among the four populations (Fig. 5*J*,*K*; Deviation, Kruskal–Wallis test; PC1, *n* = 104, df = 3, *H* = 1.38, *p* = 0.71; PC2, *n* = 104, df = 3, *H* = 0.53, *p* = 0.91). Vector deviations in this full-shuffle control were clearly larger than those in the original data without shuffle (Wilcoxon rank-sum test, *n* = 52; PC1, DS, *W* = 205, *p* = 0.005; VS, *W* = 112, *p* < 0.001; cOFC, *W* = 65, *p* < 0.001; mOFC, *W* = 177, *p* < 0.001; PC2, DS, *W* = 310, *p* = 0.353; VS, W = 117, *p* < 0.001; cOFC, W = 135, *p* < 0.001; mOFC, *W* = 238, *p* = 0.028). In this full-shuffle control, eigenvectors were directed in various directions compared with those in the shuffled condition 1 (Fig. 5*F*,*J*). Thus, these shuffle procedures appropriately evaluated the significance of our population findings.

**Figure 7.**
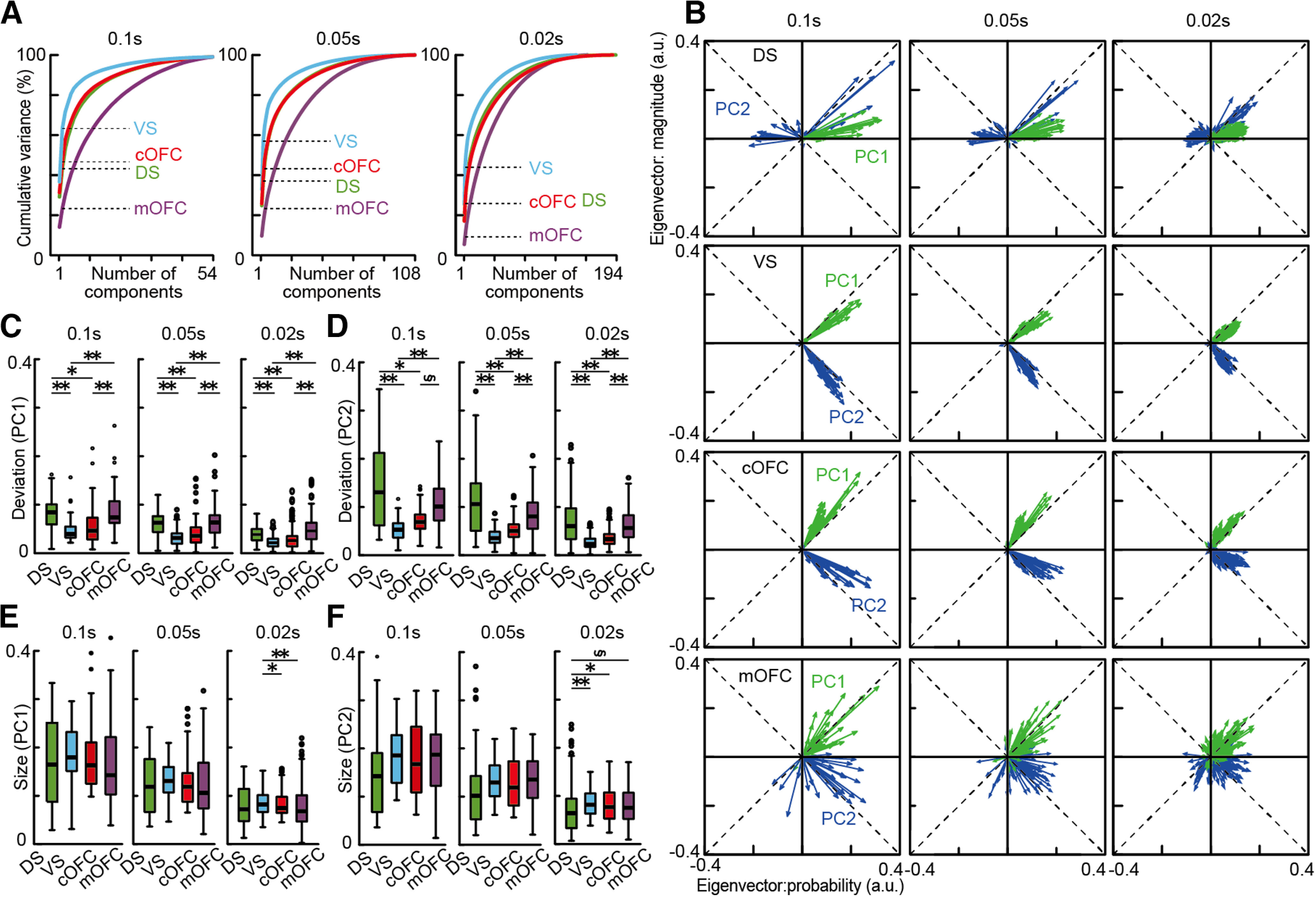
Effects of the analysis window size on the PCA. ***A***, Cumulative variances explained by PCA in the four neural populations. Dashed lines indicate the percentages of variance explained by PC1 and PC2 in each neural population. The sizes of the analysis window are 0.1, 0.05, and 0.02 s, respectively. ***B***, Overlay plots of series of eigenvectors in the four neural populations. Eigenvectors for PC1 and PC2 are shown. The analysis window size is 0.1, 0.05, and 0.02 s, respectively. a.u., Arbitrary units. ***C***, Box plots of vector deviation from the mean vector estimated in each neural population are shown for the PC1. ***D***, Same as ***C***, but for the PC2. ***E***, Box plots of vector size estimated in each neural population are shown for the PC1. ***F***, Same as ***E***, but for the PC2. In ***C–F***, asterisks indicate statistical significance between two neural populations using Wilcoxon rank-sum test with Bonferroni correction for multiple comparisons [statistical significance at **p < 0.01, *p < 0.05, and §0.05 < *p* < 0.06 (close to significance), respectively].

Next, we examined whether eigenvector size differed among the four neural populations, which represents the extent of neural modulation by probability and magnitude in each neural population as an arbitrary unit. The eigenvector size was not significantly different (Fig. 5*D*, left; PC1, Kruskal–Wallis test, *n* = 104, df = 3, *H* = 2.62, *p* = 0.45, right; PC2, *n* = 104, df = 3, *H* = 4.76, *p* = 0.19), but it strongly depended on the temporal resolution (Fig. 7). The eigenvector size decreased with the analysis window size (Fig. 7*B*,*E*,*F*), although all the results and conclusions described above were maintained across the window sizes (Fig. 7*A–D*). The decrease in the eigenvector size could be because signal-to-noise ratios generally decrease when the window size decreases. These effects were observed as a decrease in PCA performance (Fig. 7*A*) and percentages of neural modulations in the conventional analyses (Fig. 2*M–P*). Note that we did not find any significant difference in the vector size compared with shuffle controls in each neural population (Fig. 5*D*,*H*,*L*; *p* > 0.05 for all cases).

Collectively, these observations suggest a possibility that the probability and magnitude of rewards could be detected and integrated within the activity of the cOFC and VS neural populations as the expected value and risk–return signals in a stable state, at least considering the four brain regions that have been thought as key components of the reward system of the brain.

### Temporal structure of neural population signals

**Figure 8.**
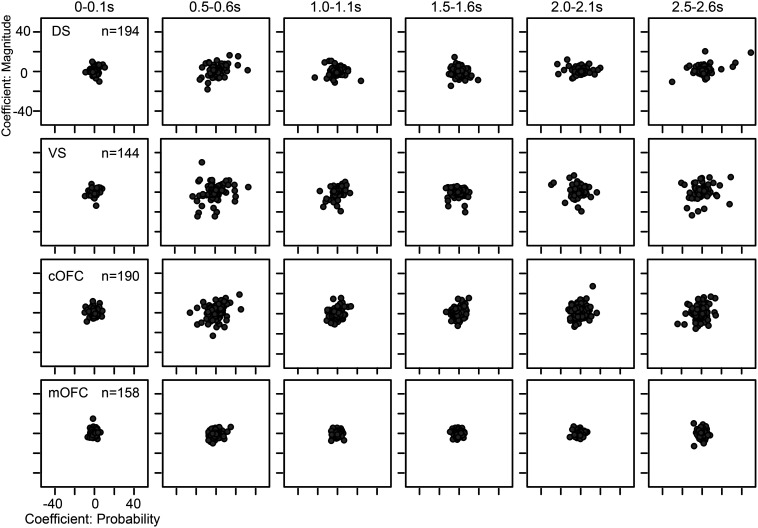
Neural modulation patterns as regression coefficients in four neural populations. Plots of regression coefficients for the probability and magnitude of rewards estimated for all neurons in the DS, VS, cOFC, and mOFC. Regression coefficients when using a 0.1 s analysis window are shown every 0.5 s (0–0.1, 0.5–0.6, 1.0–1.1, 1.5–1.6, 2.0–2.1, and 2.5–2.6 s).

**Figure 9.**
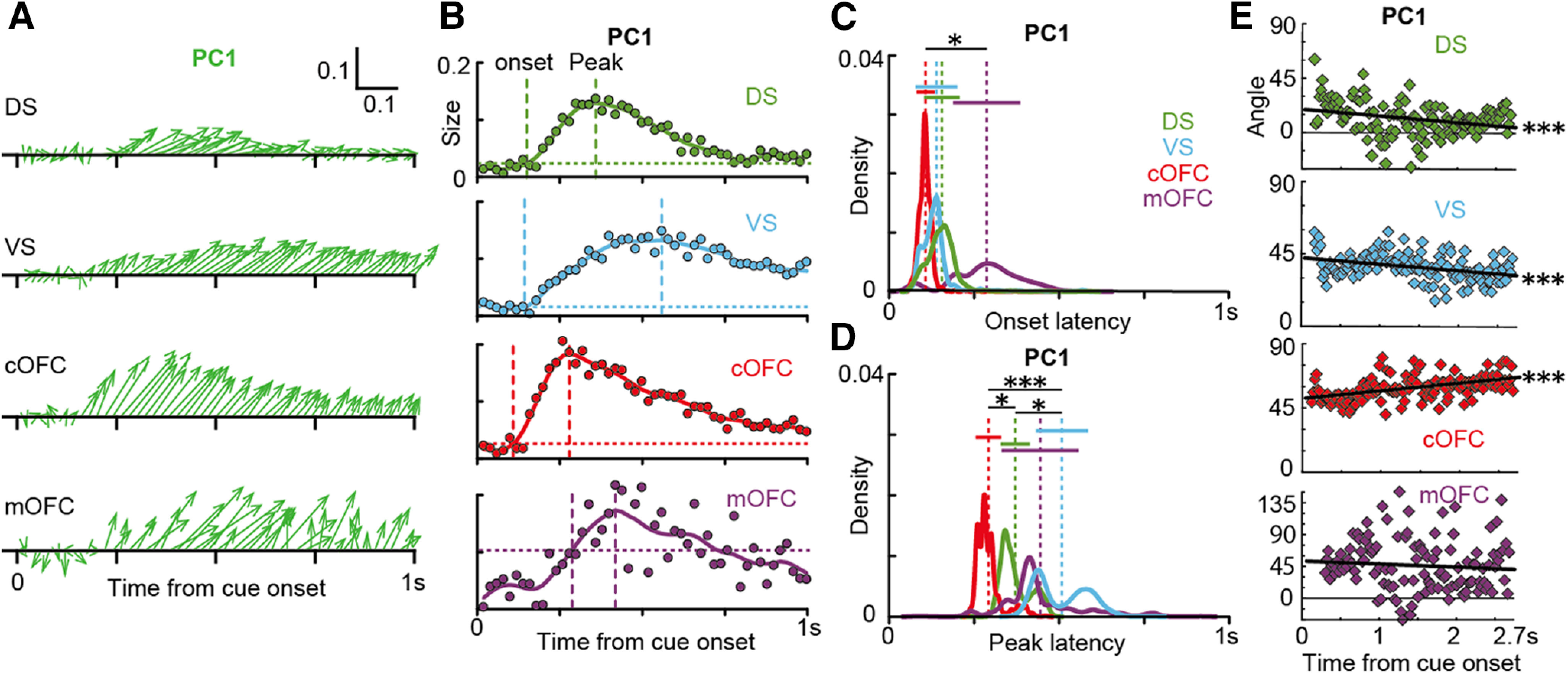
Gradual and sharp evolutions of neural population signals in the VS and cOFC. ***A***, Plots of eigenvector time series for PC1 in 0.02 s analysis windows shown in a sequential order during 1 s after cue onset. Horizontal and vertical scale bars indicate the eigenvectors for probability and magnitude in arbitrary units, respectively. ***B***, Plots of the time series of vector size during 1 s after cue onset. Horizontal dashed lines indicate 3 SDs of the mean vector size during the baseline period, a 0.3 s time period before cue onset. Solid colored lines indicate interpolated lines using a cubic spline function to provide a resolution of 0.005 s. Vertical dashed lines indicate the onset (left) and peak (right) latencies for changes in vector sizes. ***C***, Probability densities of onset latencies for the four neural population signals. Probability densities were estimated using bootstrap resamplings. Vertical dashed lines indicate means. Horizontal solid lines indicate bootstrap SEs. ***D***, Same as ***C***, but for peak latencies of the four neural population signals. ***E***, Plots of time series of vector angle from the detected onset to the onset of outcome feedback. Solid black lines indicate regression slopes. In ***C*** and ***D***, asterisks indicate statistical significance estimated using bootstrap resamplings (statistical significance at ***p < 0.001 and *p < 0.05, respectively). In ***E***, triple asterisks indicate a statistical significance of the regression slope at *p* < 0.001. Data for PC2 are not shown.

Although stable signals were observed in the cOFC and VS neural populations above, the extent of neural modulations changed throughout a trial (Fig. 8). To characterize temporal aspects of the VS and cOFC neural populations that yield expected value signals, we first compared temporal dynamics of all four neural population signals at the finest time resolution. Specifically, we compared the temporal patterns of vector changes exhibited by each neural population (Fig. 9). At the time point after cue onset when monkeys initiated the expected value computation, all four neural populations developed eigenvectors (Fig. 9*A*). The eigenvector size increased and then decreased within a second; however, the temporal patterns of this size change were different among the four neural populations. The onset latencies, detected by comparing to the vector size during the baseline period, seemed to be coincident for the cOFC, VS, and DS populations, followed by a late noisy signal in the mOFC (Fig. 9*B*). In contrast, the detected peak of vector size for each neural population seemed to appear at different times. To statistically examine these temporal dynamics at the population level, we used a bootstrap resampling technique (see Materials and Methods).

The analysis revealed no significant difference in onset latencies among the cOFC, VS, and DS populations (Fig. 9*C*; bootstrap resampling, onset latency, mean ± SD; cOFC, 107.1 ± 26.0 ms; VS, 138,7 ± 61.3 ms; DS, 155.0 ± 52.4 ms), while these signals were followed by a late noisy signal in the mOFC (287 ± 98.8 ms). In contrast, when we compared peak latencies (Fig. 9*D*), the cOFC exhibited the earliest peak (292 ± 37.5 ms), followed by the DS (371 ± 43.0 ms), the mOFC (444 ± 113.5 ms), and the VS (508 ± 76.7 ms), which exhibited the latest peak. Thus, the expected value signal sharply developed in the cOFC in contrast to the gradual development in the VS. mOFC signals were very noisy, as indicated by the large variation in the vector size during the baseline period (Fig. 9*B*, bottom; see horizontal line).

We also examined temporal changes in vector angles, which indicate how fast the stable expected value signals were evoked in the cOFC and VS (Fig. 9*E*). As observed in the time series of vector angles after detected onsets, signals carried by the VS and cOFC neural populations during the early time period were almost 45° (i.e., expected value), indicating that these two neural populations integrate probability and magnitude information into expected value just after the appearance of the numerical symbol (see intercepts of regression lines). Moreover, these two expected value signals were not the same, but rather were idiosyncratic in each neural population: a gradual and slight shift of the vector angle directed to 90° (i.e., magnitude; cOFC, Fig. 9*E*; regression coefficient, *r* = 5.31, *n* = 129, *t* = 6.04, df = 126, *p* < 0.001) or 0° (i.e., probability; Fig. 9*E*; VS, *r* = −3.91, *n* = 127, *t* = −4.16, df = 124, *p* < 0.001) was observed toward the end of cue presentation. Similar to the VS population, the DS population showed the same tendency as the angle shift (Fig. 9*E*; DS, *r* = −5.38, *n* = 127, *t* = −3.31, df = 124, *p* = 0.001). In contrast, a significant shift in vector angle was not observed in the mOFC population (*r* = −4.30, *n* = 120, *t* = −0.94, df = 117, *p* = 0.351). The signals observed in the DS and mOFC populations immediately after cue presentation were relatively close to the expected value; however, they quickly disappeared (Fig. 9*E*). These results suggest that the neural populations in both the VS and cOFC integrate probability and magnitude information into expected value immediately after cue presentation, despite their temporal dynamics being idiosyncratic for each of the two stable population signals.

### Neural population structure with multiplicative integration of probability and magnitude

**Figure 10.**
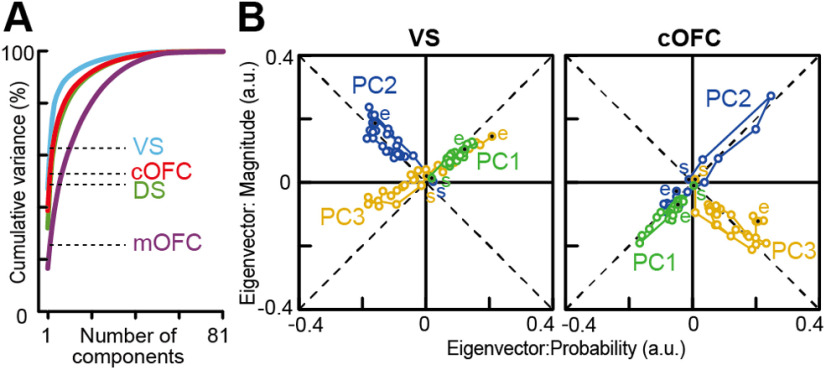
Neural population structures of the VS and cOFC with multiplicative integration of probability and magnitude. ***A***, Cumulative variance explained by PCA in the four neural populations when the state space analysis was performed with the expected value into the regression matrix. Dashed line indicates the percentage of variances explained by PC1 and PC2 in each neural population. ***B***, Plots of time series of eigenvectors connected with lines for PC1 to PC3 in the VS and cOFC. Eigenvectors during cue presentation were presented from the beginning to the end using a 0.1 s analysis window. Plots at the beginning and end are filled in black and labeled as start (s) and end (e), respectively. a.u., Arbitrary unit.

We detected the expected value signals in the VS and cOFC as a particular vector angle defined as a linear combination of probability and magnitude in their regression subspace above. This original state space analysis could not differentiate whether neural populations use linear or multiplicative integration, although the expected values assume a multiplicative combination of probability and magnitude, mathematically. Last, we examined whether these neural populations use multiplicative integration by performing an additional state space analysis, which determines whether the original neural population structure, represented as a linear combination of probability and magnitude, is unaffected by the existence of multiplicative integration (see Materials and Methods). Performance of the additional state space analysis in each population was similar to that in the original analysis (Figs. 5*A*, 10*A*). Slight increases in explained variance were observed for PC1 and PC2 (<10% in the cOFC and DS), suggesting that the neural populations in the VS and cOFC may be similarly explained by linear and multiplicative integration.

The neural population structure represented as eigenvectors was consistently observed in the VS (Fig. 10*B*, left). PC1 and PC2 signaled expected value (left, green) and risk–return (left, blue), as observed in the original analysis (Fig. 5*B*). Eigenvector directions for PC2 were flipped compared with the original ones, possibly because changes in coordinate transformation by including the expected value subspace can affect polarity determination in the component plane. Note that eigenvectors evolved after cue presentation (Fig. 10*B*, labeled with “s”) and developed toward the end of cue presentation (Fig. 10*B*, labeled with “e”) consistent with those in the original analysis (Fig. 9*A*). In contrast, the predominant eigenvectors were changed in the cOFC (Fig. 10*B*, right). Eigenvectors for both PC1 and PC2 were directed to the expected value by complimenting with each other (i.e., 45° and 225°), while the risk–return signal decreased from PC2 to PC3. This may be because a considerable degree of variance unexplained in the original analysis was added by including the expected value into the regression subspace in the cOFC. These results suggest that using linear or multiplicative integration resulted in somewhat different stable neural population structures in the cOFC.

## Discussion

Extraction of neural population dynamics is a recently developing approach for understanding computational processes implemented in the domain of cognitive and motor processing (Churchland et al., 2012; Mante et al., 2013; Chen and Stuphorn, 2015; Murray et al., 2017; Takei et al., 2017). This approach provides a mechanistic structure of neural population signals regarding temporal aspects, such as oscillatory activities during reaching (Churchland et al., 2012), coactivation patterns of spinal neurons and muscles (Takei et al., 2017), and dynamic unfolding of task-related activity during perceptual decisions (Mante et al., 2013). Here, we found that the VS and cOFC neural populations maintain the stable expected value signals at the population level (Fig. 5). This is the first mechanistic demonstration of expected value signals embedded in multiple neural populations when monkeys computed expected values from numerical symbols cueing the probability and magnitude of rewards. The temporal dynamics of these two stable neural populations are unique in the aspect of time constants (Fig. 9*B–D*) and gradual shifts of their structures (Fig. 9*E*). These results suggest that cOFC and VS compute expected values as distinct, partially overlapping processes. If monkeys are required to make an economic choice, these expected value computations must be followed by comparison and choice processes employed by the same or downstream brain regions (Raghuraman and Padoa-Schioppa, 2014; Chen and Stuphorn, 2015; Zhou et al., 2019; Yoo and Hayden, 2020).

### Two idiosyncratic expected value signals in the cOFC and VS

State space analysis can detect both stable (Murray et al., 2017) and flexible (Mante et al., 2013) neural signals at the population level. In the present study, the expected value signals observed in the VS and cOFC were similarly stable in terms of vector angle fluctuation but significantly different in temporal aspects (Fig. 9). These signal properties indicate that information processing in these two brain regions was not the same. For example, the fast cOFC signal may reflect the calculation of expected values from the probability and magnitude symbols, such as mental arithmetic, while the slow VS signal may reflect a secondary process to maintain the calculated expected value information. It is also possible that the fast cOFC signal may have reflected expected value signals integrated elsewhere (e.g., the amygdala). It is known that the frontostriatal projection plays a large role in a variety of cognitive functions anatomically (Alexander et al., 1986; Haber and Knutson, 2010). Since the cOFC projects to the VS, these two processes must act cooperatively through the cortico-basal ganglia loop. Indeed, these population signals were similar in terms of the heterogeneous signals carried by each individual neuron (Fig. 2*J*,*K*) throughout the task trial (Fig. 2*N*,*O*). However, these two expected value signals were unambiguously distinctive in terms of their time course (Fig. 9*B–D*) and gradual shift (Fig. 9*E*). Therefore, the cOFC and VS may compute expected values within each cortical and striatal local circuits in a cooperative manner.

Our results are consistent with those of human imaging studies, in which the activity in the VS and cOFC represented value-related signals (O'Doherty et al., 2004; Yan et al., 2016; Noonan et al., 2017), but not with the evidence that value signals exist in the human ventromedial prefrontal cortex (vmPFC; Tom et al., 2007; Levy and Glimcher, 2011), which includes the mOFC. The reasons for why the mOFC showed very weak signals related to all aspects of expected value (Figs. 2*L*, 5*B*) is unclear. One possibility for this inconsistency may be interspecific differences between human and nonhuman primates in the orbitofrontal network (Wallis, 2011). The mOFC is a part of the vmPFC, but the comparison between human and macaque monkeys remains elusive. Another possibility is that the vmPFC is not involved in simple information processing, such as the association between cues and outcomes, but is involved in more complicated behavioral contexts for making economic decisions (Yamada et al., 2018) and setting of mood (Ongür and Price, 2000).

### Fluctuating signals in the DS and mOFC

Fluctuating signals were observed in the DS and mOFC because of the instability or weakness of the signals (Fig. 5). The mOFC signal would not be completely meaningless, since the PCA performance in the mOFC population was better than in shuffle controls (Fig. 6). However, the signal carried by the mOFC population was weak (Fig. 2*L*), indicating that the eigenvector fluctuation in the mOFC population reflects weak signal modulations by probability and magnitude. In contrast, PCA performance in the fluctuating DS population was equivalent to that in the cOFC population (Fig. 5*A*), where a stable expected value signal appeared. Moreover, considerable modulation of DS neural activity was observed in conventional analyses (Fig. 2*I*,*M*). Thus, the fluctuating DS signal must reflect a functional role played by the DS neural population in detecting and integrating probability and magnitude, which is related to some controls of actions (Balleine et al., 2007). The DS signal fluctuated with a significant shift directing probability, but the initial signal was relatively close to expected values (Fig. 9*E*, top), which is similar to the instantaneous expected value signals observed in the mOFC (Fig. 9*E*, bottom). These observations imply that the expected value computations might be distributed in the reward circuitry. The consistent direction of the shift between VS and DS populations implies that striatal neural populations may prefer probabilistic phenomena (Pouget et al., 2013; Ma and Jazayeri, 2014), whereas the cOFC neural population may prefer magnitude, which is a continuous variable.

### Expected value signals and economic choices

Economic choices seem to be composed of a series of processes, such as expected value computation, followed by value comparison, and then choice among options. Recent findings suggest that these computations may or may not be discrete/continuous and could overlap (Chen and Stuphorn, 2015; Yoo and Hayden, 2020). Because we used a single-cue task, the observed signals solely reflect the integration of probability and magnitude. In the last 2 decades, neural correlates of probability and/or magnitude have been extensively reported in a diverse set of brain regions (O'Doherty, 2014), mostly during economic choice tasks without reflecting on their underlying dynamics. These distributed signals may support the possibility that expected value computation occurs in wider brain regions as a network, although they are likely to reflect an array of alternative non-value-related processes (O'Doherty, 2014), such as motor responses and choice processes. Although signals in the DS and mOFC fluctuated (Fig. 5*B*), they were relatively close to expected values at the beginning of cue presentation (Fig. 9*A*,*E*), suggesting that widespread evolution of expected value signals might occur through a reward circuitry at the beginning when monkeys process the integration.

### Significance of population signals revealed by our analysis

State space analysis reveals temporal structures of neural populations in multidimensional space for both cognitive tasks (Murray et al., 2017) and motor tasks (Churchland et al., 2012; Takei et al., 2017). However, interpretation of the extracted population structure depends on the method used (Elsayed and Cunningham, 2017). In the present study, we did not seek to determine the population structure as a trajectory in neural state space, as performed in previous studies. Instead, we aimed to detect the main features underscoring the population structure in the space of probability and magnitude that compose expected value. For this purpose, stability of the regression subspace is critical. We elaborately projected neural firing rates into the regression subspace by preparing a completely orthogonal data matrix in our task design. Moreover, two shuffled controls revealed the significance of our state space analysis. In the full-shuffled control, eigenvectors directed all dictions, because neural modulation structures were entirely destroyed (Fig. 5*J*). In the partially shuffled control (condition 1), the maintained temporal structure occasionally yields some subtle modulation structures through a trial because of the random allocation of neural activity to probability and magnitude (Fig. 5*F*). Thus, our state space analysis is informative on whether and how expected value signals are composed of the probability and magnitude moment by moment as a series of eigenvectors.

### Conclusions

A dynamic integrative process of probability and magnitude is the basis for the computation of expected values in particular brain regions (i.e., the cOFC and VS). The existence of neural population signals for expected values is consistent with the expected value theory, whereas the coexistence of risk signals, which has been shown (O'Neill and Schultz, 2010) with returns (Figs. 3, 5*B*), may reflect a behavioral bias for risk preferences, a phenomenon observed across species (Stephens and Krebs, 1986; Yamada et al., 2013a). The sharp and slow evolution of expected value signals in the cOFC and VS, respectively, suggests that each brain region has a unique time constant in the expected value computation. When monkeys perceive probability and magnitude from numerical symbols, learned expected values may be computed and recalled through the OFC–striatum circuit (Hirokawa et al., 2019), along with other networks that may also instantaneously process this computation. Our results indicate that the expected value signals observed in population ensemble activities are compatible with the framework of dynamic systems (Churchland et al., 2012; Mante et al., 2013).
